# Simple bedside score to optimize the time and the decision to initiate appropriate therapy for carbapenem-resistant *Enterobacteriaceae*

**DOI:** 10.1186/s12941-015-0088-y

**Published:** 2015-06-04

**Authors:** Violet Leibman, Emily T. Martin, Ruthy Tal-Jasper, Leonti Grin, Kayoko Hayakawa, Coral Shefler, Tal Azouri, Tamir Kaplansky, Moran Maskit, Tsilia Lazarovitch, Ronit Zaidenstein, Keith S. Kaye, Dror Marchaim

**Affiliations:** Sackler School of Medicine, Tel-Aviv University, Tel-Aviv, Israel; Department of Epidemiology, University of Michigan School of Public Health, Ann Arbor, MI USA; Unit of Infectious Diseases, Assaf Harofeh Medical Center, Zerifin, 70300 Israel; Division of Infectious Diseases, Detroit Medical Center, Wayne State University, Detroit, MI USA

**Keywords:** CRE, KPC, ESBL, Prediction score, Nosocomial infection, Multidrug resistant

## Abstract

**Background:**

Epidemiological characteristics of patients with bloodstream infections (BSI) due to extended-spectrum β-lactamase producing (ESBL) and carbapenem-resistant (CRE) strains are often similar. Mortality rates for CRE BSI are 70 %, and mean time to initiation of appropriate therapy is ~5 days. A bedside score was developed to differentiate CRE-BSIs from ESBL-BSIs, in order to help decrease the time to initiation of appropriate therapy for CRE and mortality rates.

**Findings:**

Score was developed based of data (2007–2010) abstracted from charts of adult patients from Assaf Harofeh Medical Center (AHMC, Zeriffin, Israel), and validated on a cohort of patients from Detroit Medical Center (DMC, MI, USA). A multivariate model for presence of CRE was generated. A clinical prediction score and ROC curve was derived. 451 patients with ESBL BSIs (285 from AHMC and 166 from DMC) and 74 patients with CRE BSIs (58 from AHMC and 16 from DMC) were included. The prediction score included chemotherapy in the past 3 months (19 points), presence of foreign invasive devices (10 points), no peripheral vascular disease (10 points), reduced consciousness or cognition at time of acute illness (9 points), time in hospital prior to BSI ≥ 3 days (7 points), and age younger than 65 years (6 points). A score of ≥32 to define “high CRE risk” had sensitivity of 59 %, specificity of 76 %, PPV of 34 % and NPV of 90 %.

**Conclusions:**

The score’s 90 % NPV implies it could reduce un-necessary (and toxic) empiric use of anti-CRE therapeutics, but this should be studied prospectively and on broader populations in order to test its potential role in reducing mortality.

Antimicrobial resistance to broad-spectrum agents among the commonest enteric pathogens (e.g. *Escherichia coli*, *Klebsiella pneumoniae*) had become endemic in many regions worldwide [1]. Both extended-spectrum β-lactamase producing Enterobacteriaceae (ESBL) and carbapenem-resistant Enterobacteriaceae (CRE) are now frequently encountered nosocomial pathogens, but there are multiple resemblances in their epidemiological features [1, 2]. While confronted with severely septic patients, in these endemic facilities, it is often challenging for physicians to tailor the most suitable empiric regimen to patients, when Enterobacteriaceae bloodstream infection (BSI) is suspected [3]. Carbapenems are still considered the agents of choice for ESBL BSIs, but are ineffective (when given alone) against CRE [3]. Since time to initiation of appropriate therapy is the strongest modifiable independent predictor for mortality in severe sepsis [4], physicians need to act fast in order to impact patients’ outcomes. However, the mean number of hours to initiation of appropriate therapy for patients with CRE is 120 ± 23 h, i.e. 5 days [5], mainly due to delays in current routine practices for CRE determination in microbiology laboratories [1]. Therefore, it is not surprising that the attributable mortality rate among patients with CRE BSIs is 70 % [6]. Physicians are reluctant to empirically prescribe anti-CRE agents (for severe invasive infection, i.e. BSI, polymixins are frequently and practically the only remaining appropriate agents) due to: 1) concerns pertaining to induction of resistance to the few remaining therapeutic options that are still available [7], 2) High rate of toxicities associated with polymixins use, and 3) scant controlled scientific data, pertaining to polymixins efficacy and pharmacokinetics properties in patients with CRE BSI [8].

An easy to calculate score, with high performance, based solely on parameters readily available bedside to attending clinicians, is needed in order to direct prescribers in their management of severely septic patients in the hospital settings. Prior attempts to develop such a score by our group, had few limitations, since it was based on only 16 CRE BSI cases during initial CRE emergence into the region, when endemicity was not yet established [9]. Our current study aims were to 1) develop a different bedside score, based on more cases and from a ‘stable’ endemic CRE region, in order to help physicians quantify the likelihood for BSI caused by CRE as opposed to ESBL, and 2) validate the score on a different cohort of patients from a distinct geographic region, that was initially studied in our first attempt for score’s development [9].

## Findings

The Assaf Harofeh Medical Center (AHMC) is an 813-bed academic tertiary facility in the southern-central part of Israel. CRE is endemic in this region [1]. Score validation was executed at the Detroit Medical Center (DMC) health system in Southeast Michigan, which consists of 2200 inpatient beds. The Institutional Review Boards of AHMC, DMC and Wayne State University approved the study prior to its initiation. The study cohort consisted of hospitalized patients with unique episodes of bacteremia who met the following inclusion criteria: bloodstream infections (BSI) caused by either CRE or ESBL-producing *Enterobacteriaceae* (monomicrobial isolations), from calendar years 2007–2010, who, on the date of culture, had severe sepsis, septic shock, or multi-organ failure. Variables collected for each patient included: 1) demographics; 2) co-morbidities; 3) recent (3 months) exposures to antibiotics; and 4) recent (3 months) exposures to various healthcare-associated environments and procedures. Bacteria were identified and susceptibilities were determined in accordance with Clinical and Laboratory Standards Institute criteria [10]. Carbapenemase production screening was conducted for *Enterobacteriacea* which were resistant to one or more third generation cephalosporins and had elevated ertapenem MIC of ≥2 μg/dL, with the modified Hodge test [10]. ESBL production was determined automatically and validated with disc diffusion tests [10]. A clinical prediction score was developed through construction of a multiple regression model for predictors of CRE BSI compared to ESBL BSI. The score was derived from the final model by multiplying the regression coefficients by a factor of 10. Score performance was assessed by calculation of sensitivity, specificity, positive predictive value (PPV), negative predictive value (NPV), and associated 95 % C.I. A receiver operating characteristic (ROC) curve was generated and area under the curve (AUC) was tested against a null AUC of 0.5.

**Fig. 1:**
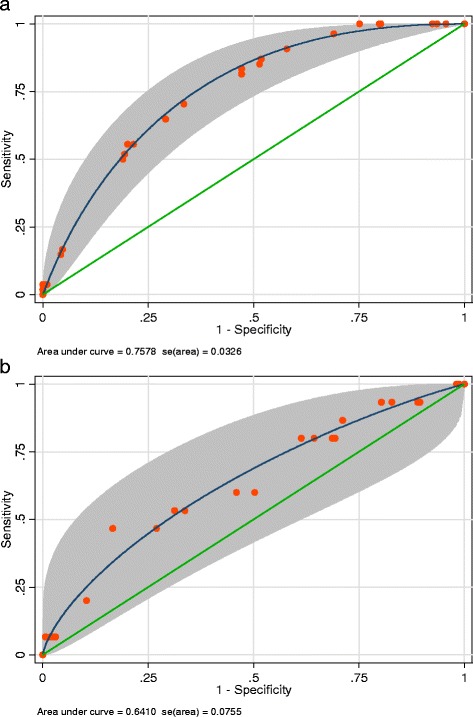
**a**: Receiver Operating Characteristic (ROC) curve of score to predict bloodstream infection (BSI) due to carbapenem-resistant Enterobacteriaceae (CRE) as opposed to extended-spectrum β-lactamase producing Enterobacteriaceae (ESBL), Assaf Harofeh Medical Center. **b**: ROC curve of the score developed at Assaf Harofeh Medical Center to predict bloodstream infection due to CRE as opposed to bloodstream infection due to ESBL-producing *Enterobacteriaceae* at Detroit Medical Center

Overall, 343 patients met inclusion criteria, including 285 patients with BSIs due ESBL-producing *Enterobacteriaceae* and 58 patients with BSIs due to CRE. The final multivariable model of predictors for CRE BSI as compared to ESBL BSI among hospitalized adult patients at AHMC is displayed in Table 1. Based on this model, a prediction score was developed (Table 1). The score had an area under ROC curve (AUC) of 0.75 (CI-95 % 0.69–0.82) (Fig. 1a). A score of ≥32 to define “high CRE risk” had sensitivity of 59 % (95 C.I. 45, 71 %), specificity of 76 % (95 C.I. 71, 81 %), PPV of 34 % (95 C.I. 25, 44 %) and NPV of 90 % (95 C.I. 86, 94 %). Using a cut-point of 32 points or higher to define CRE high risk was then validated on a cohort of patients from DMC: 166 with ESBL BSIs and 16 patients with CRE BSIs. The score had an area under ROC curve of 0.64 (Fig. 1b), sensitivity of 56 % (95 C.I. 30, 80 %), specificity of 65 % (95 C.I. 57, 72 %), PPV of 13 % (95 C.I. 6, 24 %) and NPV of 94 % (95 C.I. 88, 98 %). In a clinical setting, the score would thus be calculated by adding the number of points for each relevant patient condition (Table 1). Patients with a score less than 32 would be considered low risk for CRE, as opposed to ESBL.

**Table 1:** Univariable analyses and multivariable model of carbapenem-resistant Enterobacrteriaceae (CRE) bloodstream infections (BSI) compared to extended-spectrum β-lactamase producing Enterobacteriaceae (ESBL) BSI: adjusted associations and final score values

	Derivation Cohort (AHMC)					Validation Cohort (DMC)	
Variable	ESBL, N (%) (*N* = 285)	CRE, N (%) (*N* = 58)	OR (95 % CI); *p*-value	AOR ^a^ (95 % C.I.); *p*-value	Score	ESBL, N (%) (*N* = 166)	CRE, N (%) (*N* = 16)
Male gender	169 (59)	29 (50)	0.69 (0.39, 1.21); 0.19			91 (55)	7 (44)
Age <65 years	47 (17)	16 (28)	1.9 (1.00, 3.71) 0.05	1.8 (0.81, 3.92); 0.16	6	78 (47)	5 (31)
LTCF residency^b^	107 (38)	14 (25)	0.53 (0.28, 1.02); 0.06			96 (58)	13 (81)
Recent (3 months) hospitalization or LTCF stay	241 (85)	45 (78)	0.63 (0.32, 1.27); 0.20			100 (60)	14 (88)
Chronic hemodialysis	15 (5)	4 (7)	1.17 (0.40, 3.40); 0.78			33 (20)	5 (31)
Deteriorated functional status at admission	223 (79)	37 (67)	0.56 (0.30, 1.06); 0.07			105 (63)	14 (88)
Congestive heart failure	86 (31)	15 (26)	0.80 (0.42, 1.51); 0.48			63 (38)	8 (50)
Diabetes mellitus	115 (40)	22 (38)	0.90 (0.51, 1.61); 0.73			86 (52)	13 (81)
Chronic renal failure^c^	97 (34)	15 (26)	0.66 (0.35, 1.25); 0.21			68 (41)	10 (63)
No PVD^d^	218 (77)	50 (86)	1.8 (0.83, 4.07); 0.14	2.7 (1.06, 7.02); 0.04	10	134 (81)	13 (81)
Any neurological disease (including past cerebral events)	157 (55)	27 (47)	0.7 (0.4, 1.3; 0.2			68 (41)	12 (75)
Past or present (active) malignancy	62 (22)	14 (25)	1.2 (0.6, 2.2; 0.7			32 (19)	2 (13)
Hemiplegia or paraplegia	49 (17)	12 (21)	1.27 (0.63, 2.58); 0.50			25 (15)	7 (44)
Chemotherapy in the past 3 months	7 (2)	6 (10)	4.53 (1.46, 14.0); 0.009	6.8 (1.9–24.7); 0.003	19	5 (3)	1 (6)
Immunosuppression^e^	51 (18)	17 (29)	1.88 (0.99, 3.57); 0.05			28 (17)	5 (31)
Any use of antibiotics in the preceding 3 months	214 (77)	52 (90)	2.6 (1.1, 6.4); 0.03			112 (68)	14 (88)
Recent (6 months) invasive procedure^f^	120 (43)	33 (59)	1.9 (1.1, 3.4); 0.03			12 (86)	15 (100)
Intensive care unit stay at infection onset	151 (53)	39 (67)	1.82 (1.00, 3.30); 0.05			67 (40)	10 (63)
Permanent foreign invasive devices^g^	145 (52)	44 (77)	3.17 (1.64, 6.15); 0.001	2.5 (1.2–5.2); 0.02	10	115 (70)	13 (81)
Reduced consciousness and/or cognition at time of acute illness	151 (53)	40 (73)	2.33 (1.23, 4.41); 0.009	2.5 (1.1–5.6); 0.02	9	100 (60)	13 (81)
Severe sepsis / septic shock / multiorgan failure at culture date	133 (48)	36 (66)	2.1 (1.1, 3.8); 0.02			43 (26)	4 (29)
Length of hospital stay at BSI^h^ onset >3 days	134 (47)	44 (76)	2.85 (1.49, 5.43); 0.001	1.9 (0.9–4); 0.08	7	61 (37)	11 (73)

^a^ *AOR* adjusted odds ratio. ^b^Long-term care facility. ^c^Serum creatinine > 1.5 mg% at baseline. ^d^Peripheral Vascular Disease. ^e^Immunosuppression include any one of the following conditions at illness onset: neutropenia (<500 cells/mm^3^), glucocorticoid / steroid use in the past month, chemotherapy in the past 3 months, radiotherapy in the past 3 months, HIV, bone marrow or solid organ transplantation, or anti-TNF-α therapy in past 3 months (e.g. infliximab, adalimumab, certolizumab pegol, golimumab, etanercept). ^f^Include any type of surgery, endoscopy, percutaneous intervention. ^g^An invasive foreign device that was in place at least 48 h prior to ESBL or CRE isolation. Examples: tracheotomy, any feeding tubes, tunneled central lines, silicon-based urinary catheters, orthopedic external fixators, implanted defibrillator, pacemaker, and drains of any sort. Prosthetic heart valve or internal prosthetic joints were not considered a permanent foreign invasive devise. ^h^bloodstream infection.

This study is our first phase in trying to develop a score that can shorten the time to institution of appropriate therapy in patients with CRE BSIs, in the hope that eventually this would lead to reductions in CRE mortality rates which are reported to be ~70 % [6]. We know that appropriate therapy for CRE infections is delayed by ~5 days [5], and that delay of instituting appropriate therapy in severe sepsis is the strongest independent predictor for mortality [4]. Therefore, apart from improving rapid diagnostic techniques, among other optional paths, a prediction score with high performance could theoretically have a valuable role in such clinical scenarios. However, clinicians are reluctant to use prediction scores in their routine clinical practice. The score has to be simple, based only on parameters that could easily be extracted while attending the patient and reviewing the chart bedside. This is our second step in our eventual goal of developing a scientific reliable CRE BSI score for adult hospitalized patients [9]. The score was re-developed using retrospective clinical data from an endemic CRE region, constituting relatively high numbers. However, some of the score components might still be unique features of the epidemiology at AHMC. The relative low performances of this suggested score, suggest larger cohorts from various endemic regions should be studied in the future. The imperfect diagnostics of CRE and ESBL production can results classification biases and should prompt consideration.

Some might argue that the clinical dilemma in today’s era for attending clinicians practicing in tertiary facilities is a bit broader. For severely septic patients in the hospital setting, instituting early broad spectrum antimicrobial agents is the common standard of care [3]. For the Gram-negative bacilli (GNB) treatment arm that should be instituted, practitioners need to choose between empiric coverage of “only” multidrug resistant (MDR) GNB isolates (e.g. ESBL-producing Enterobacteriaceae, broad-spectrum cephalosporin’s resistant *Acinetobacter baumannii* and *Pseudomonas aeruginosa*), as opposed to empiric coverage for extensively-drug resistant (XDR) GNBs as well (e.g. CRE, and carbapenem-resistant *A. baumannii* and *P. aeruginosa*) [3]. Instead of developing a “CRE score”, which addresses only the Enterobacteriaceae angle, one might argue that developing a “XDR score” would be more clinically applicable. We look at this score as an initial step. Our future goals are to develop and validate both potential scores prospectively, on larger cohorts of patients, from distinct geographic locations, where both MDR and XDR GNBs are endemic in the hospital settings. This analysis is a crucial and valuable step in our effort to develop eventually a score with high performances, which could lead eventually to reductions in CRE mortality rates, while avoiding the un-necessary empiric use of broad spectrum and toxic agents (e.g. polymixins).

## Availability of supporting data

The data set(s) supporting the results of this article is (are) included within the article (and its additional file(s)).
